# Disparities in quality of life, social distress and employment outcomes in Australian cancer survivors

**DOI:** 10.1007/s00520-022-06914-w

**Published:** 2022-03-12

**Authors:** Victoria M. White, Karolina Lisy, Andrew Ward, Eli Ristevski, Melanie Clode, Kate Webber, Jon Emery, Maarten J. Ijzerman, Nina Afshar, Jeremy Millar, Peter Gibbs, Sue Evans, Michael Jefford

**Affiliations:** 1 School of Psychology, Faculty of Health, Deakin University, Geelong, VIC Australia; 2 Cancer Council Victoria, Melbourne, VIC Australia; 3 Australian Cancer Survivorship Centre, Peter MacCallum Cancer Centre, Melbourne, VIC Australia; 4 Department of Health Services Research, Peter MacCallum Cancer Centre, Melbourne, VIC Australia; 5 Sir Peter MacCallum Department of Oncology, The University of Melbourne, Melbourne, VIC Australia; 6 The Social Research Centre, Melbourne, VIC Australia; 7 Monash Rural Health, Monash University, Warragul, VIC Australia; 8 School of Clinical Sciences, Monash University, Clayton, VIC Australia; 9 Department of Oncology, Monash Health, Clayton, VIC Australia; 10 Department of General Practice and Centre for Cancer Research, University of Melbourne, Melbourne, Australia; 11 Centre for Epidemiology and Biostatistics, School of Population and Global Health, The University of Melbourne, Melbourne, Australia; 12 Alfred Health Radiation Oncology, Alfred and LaTrobe Regional Hospital, Melbourne, VIC 3004 Australia; 13 School of Public Health and Preventive Medicine, Monash University, Melbourne, VIC 3004 Australia; 14 Department of Medical Oncology, Western Health, St. Albans, Victoria, Australia; 15 Personalised Oncology Division, Walter and Eliza Hall Institute of Medical Research, Parkville, VIC Australia

**Keywords:** Cancer, Cancer survivors, Patient-reported outcomes, Quality of life, Social difficulties, Employment, Disparities

## Abstract

**Purpose:**

To examine how socio-demographic, comorbidities and information needs influence quality of life (QoL) outcomes of survivors of breast, colorectal, or prostate cancer, non-Hodgkin lymphoma or melanoma.

**Methods:**

Cross-sectional postal survey with eligible participants identified through a population-based cancer registry. QoL outcomes were assessed by EQ-5D-5L, social difficulties index (SDI) and, for those employed at diagnosis, current employment. Regression analyses explored associations between outcome variables and cancer type, age, time since diagnosis, residential location, socio-economic disadvantage, comorbidities and unmet information needs. Mediation analyses examined whether comorbidities and information needs explained relationships between outcome variables and socio-economic disadvantage.

**Results:**

2115 survivors participated. Mean EQ-5D-5L scores (mean = 0.84) were similar to population averages and SDI scores were low for the entire sample (mean = 3.80). In multivariate analyses, being aged over 80, greater socio-economic disadvantage, comorbidities and unmet information needs decreased EQ-5D-5L scores. Higher SDI scores were associated with socio-economic disadvantage, comorbidities and unmet information needs. Not being employed was associated with being aged over 50, more comorbidities and socio-economic disadvantage. Comorbidities but not information needs partially mediated the impact of socio-economic disadvantage on EQ-5D-5L and SDI accounting for 17% and 14% of the total effect of socio-economic disadvantage respectively. Neither comorbidities nor information needs mediated the association between socio-economic disadvantage and employment outcomes.

**Conclusions:**

To improve quality of life, survivorship care should be better tailored to address the needs of individuals given their overall health and impact of comorbidities, their age and type of cancer and not simply time since diagnosis.

**Supplementary Information:**

The online version contains supplementary material available at 10.1007/s00520-022-06914-w.

## Background

With better treatments and earlier detection, an increasing number of people are surviving cancer, with recent estimates suggesting seven out of every 10 people diagnosed with cancer in Australia will survive 5 or more years [1]. While most people recover well after cancer treatment, many experience a range of physical, emotional, psychological, social, financial and practical challenges [2–4]. Some survivors are at risk of developing complications many years after finishing cancer treatments [4] and survivors are at risk of developing other cancers [4, 5]. A number of socio-demographic factors have been associated with variation in outcomes for cancer survivors. Cancer survival is known to vary by socio-economic advantage/disadvantage [6–11] with this difference partly explained by variations in stage at diagnosis and treatment differences [6]. Recent evidence suggests that psychosocial factors including living alone, being unmarried and higher levels of depression can also negatively impact survival [12, 13]. Identifying whether socio-economic and demographic disparities also influence QoL outcomes for cancer survivors and the potential drivers of any differences can inform the type of survivorship care that should be directed to different cancer survivors to help ensure equitable outcomes [14].

Few studies have utilised a population-based sample to assess associations between socio-demographic factors such as socio-economic position, rurality and comorbid conditions and patient-reported outcomes, including QoL. Results in this area have been variable with associations differing by cancer type and outcomes. For instance, in a population-based study from the UK, QoL was significantly worse for those from the most deprived areas, compared to those from the most affluent areas for breast and prostate cancer but not non-Hodgkin lymphoma or colorectal cancer [15]. While similar inverse associations between socio-economic position and QoL have been found in other studies for breast and prostate cancer [16], different patterns of association have been reported for colorectal cancer [17–19]. While studies examining the association between socio-economic position and QoL generally control for disease, treatment and other demographic factors, controlling for presence of comorbid conditions is variable. Multiple studies have shown a consistent inverse association between number of comorbid conditions and QoL [17, 20–22]. As socio-economic position is inversely related to presence of comorbidities, associations between socio-economic position, comorbidity and QoL in cancer survivors can be confounded. Understanding sources of disparities in survivorship outcomes would enable efforts to minimise variation, improve outcomes and inform models of care.

The provision of information to assist survivors to manage their post treatment, health and wellbeing is a key component of quality survivorship care [23] yet many survivors report unmet information, supportive and emotional needs [24–28]. QoL outcomes are inversely associated with levels of unmet information and supportive care needs [23, 29, 30]. Several studies have identified disparities in the experience of unmet needs with younger, those having: a non-white race/ethnic background, comorbid conditions and lower education and income, reporting a greater number of unmet needs [23, 30].

As cancer survivorship care should seek to reduce disparities in outcomes, this study aimed to understand whether and if so how QoL outcomes (health-related QoL (HRQoL), social difficulties, employment) for cancer survivors vary by different socio-demographic factors, presence of comorbidities and information needs. Given potential confounding between socio-economic position, comorbidities and unmet information needs, we aimed to determine the direct and indirect effects of socio-economic position on QoL by exploring whether its association is mediated by comorbidity and unmet information needs. The study focuses on survivors of common cancers, three with 5-year survival rates over 90% (breast, prostate, melanoma) and two with lower (around 70%) 5-year survival (colorectal, non-Hodgkin’s lymphoma (NHL)) [31].

## Methods

### Study design

Cross-sectional survey of individuals diagnosed with five specified cancers where invited participants had a diagnosis date 1, 3 or 5 years prior to study approach.

### Subject eligibility

Potential participants were identified through the population-based Victorian Cancer Registry (VCR) that maintains a list of all people with a new diagnosis of cancer in Victoria, Australia’s second most populous state. Eligibility criteria were diagnosed with breast, prostate or colorectal cancer, melanoma or NHL approximately 1, 3 or 5 years prior to study approach, aged 18 years and over at diagnosis, and resided in Victoria at diagnosis. People were ineligible if they were deceased, had a previous cancer diagnosis or did not meet the cancer-specific morphological or histopathological criteria.

### Patient approach and consent

Following VCR standard recruitment practices, notifying clinicians of potential participants were contacted by mail to confirm eligibility. Unless clinicians indicated otherwise, eligible people were approached by mail during the month corresponding to the anniversary of their diagnosis. Reminders were sent to non-responders after 3 weeks. Invitation letters included the study information sheet, questionnaire and reply-paid envelope. A toll-free phone information service was provided. Potential participants were advised that, consent was implied by return of the questionnaire.

### Questionnaire design and content

Questionnaires developed for NHL, breast, colorectal and prostate cancer were based on UK’s National Health Service (NHS) experience of cancer care survey (with permission) [15]. Using the format, structure and topic areas covered by the NHS surveys, a new melanoma patient-reported outcome (PROs) questionnaire was developed in collaboration with an expert working group. Outcome and predictor variables were the same in each survey.

### Outcome measures

HRQoL was measured by the EQ-5D-5L scale, a standardised measure of health status which consists of five QoL domains (mobility, self-care, usual activities, pain and discomfort, anxiety and depression), with respondents selecting one of five response items in each domain to reflect their current status [32]. Australian-specific calculations were made with the nominal range of values being 0 (death) to 1 (perfect health) [33].

The social difficulties index (SDI-21) is a 21-item questionnaire used to assess everyday problems people affected by cancer may experience [34]. All items are responded to using a 4-point scale ranging from “0” = no difficulty to “3” = very much difficulty. Items assess difficulties across a range of activities, for example working, domestic chores, self-care, communicating and financial issues. Responses are summed to provide an index of difficulties with scores ranging between 0 and 44.

Participants were asked if they were working at the time of their diagnosis and their work status at the time of completing the survey. Respondents working full/part-time or casually were classified as being in paid employment at that time point.

### Predictor variables

Presence of other long-term conditions was assessed with respondents indicating if they had any of 17 listed conditions that included heart disease, diabetes, asthma and high blood pressure with an option to indicate another condition if needed (see Supplementary Table 1 for full list). The number of conditions indicated was counted to form a comorbidity index ranging from 0 (no other condition) to 8 (highest reported number of multiple conditions). Respondents indicated their need for further information (yes or no) in 10 areas (e.g. diet and lifestyle, physical activity, pain management, managing the psychological or emotional aspects of life after cancer) with responses grouped into (i) no needs, (ii) 1–3 needs or (iii) ≥ 4 needs.

The VCR provided information on the cancer diagnosis, age, gender and residential postcode. Residential postcode was used to infer socio-economic position using the relative disadvantage scale from the socio-economic indexes for areas (SEIFA) developed by the Australian Bureau of Statistics. This area-based indicator ranks each area in Australia according to its socio-economic disadvantage based on a number of indicators including unemployment, income, education and home-ownership in the area [35]. For this study, we used postcode level rankings of relative disadvantage which were grouped into deciles with low scores indicating greater disadvantage.

### Control variables

Language spoken at home (English or other), country of birth (Australia or other), residential location (metropolitan, regional, outer regional) and response to treatment were indicated by participants on the survey and controlled in analyses.

### Statistical analysis

Bivariate (chi-square and *t*-tests) and multivariate (linear or logistic regression) analyses were conducted to examine associations between the QoL outcomes (EQ-5D-5L, SDI) for all participants and for participants aged under 60 and in paid employment when diagnosed, for the outcome of not working at the time of the survey. Key predictor variables in all analyses were cancer type, time since diagnosis, gender, age, socio-economic disadvantage, comorbidities and unmet information needs. Country of birth, residential location and response to treatment were controlled in all analyses. In multivariate regression analyses, the base category was used as the reference group for all categorical predictors. Socio-economic disadvantage deciles and number of comorbidities were treated as continuous variables.

Mediation analysis aims to identify mechanisms that may explain or clarify the relationship between an independent and dependent variable. It examines whether some of the association between the independent and dependent variable arises due to their association with another variable, with the analysis testing whether this other variable (the mediator) has a significant role in the causal pathway. The effect of the independent variable through the mediator is known as the indirect effect (IE) and the effect not through the mediator is known as the direct effect (DE). In this study, mediation analyses examined whether comorbidities and unmet information needs mediated associations between socio-economic position and the three QoL indicators. Mediation analyses were conducted using the PROCESS macro (model 4) [36] for SPSS26. This macro utilises a bootstrapping procedure (sample set to *n* = 5000) to conduct unstandardised multiple linear regression to provide estimates of the direct and indirect effects of the independent variable. For outcomes, EQ-5D-5L and SDI, unstandardised beta coefficients and 95% confidence intervals (CIs) are estimated for the direct and indirect effects. For these analyses, if the 95% CIs do not include 0, the association is considered statistically significant. For the binary outcome (not in workforce), logistic regression estimated odds ratios (ORs) and their 95% CIs. In these analyses, if the 95% CI includes 1, the association is not statistically significant. The percentage of the total effect accounted for by the mediator can be estimated by dividing the IE by the total effect. All mediation models controlled for variables identified as significant in regression analyses described above.

### Ethical approval

Ethics approval was granted by the Cancer Council Victoria Human Research Ethics Committee (Project No: HREC 1307).

## Results

### Survey response rates and sample characteristics

The survey response rate overall was 45.3% (2115/4674). While response rates were slightly lower for those 5 years post diagnosis, previous work with this dataset showed no significant difference by time since diagnosis [3]. Table 1 summarises the demographic profile and self-reported comorbidities, response to treatment, information needs and symptom score of participants. There were roughly similar proportions of breast, prostate, colorectal and melanoma survivors in the sample with lowest numbers found for non-Hodgkin’s lymphoma (NHL) survivors (11%). Slightly more participants were male (53%) and the majority were under 70 years of age (Table 1). Time since diagnosis varied by cancer type with the proportion of participants 5 years post diagnosis greater for NHL (45%) and least for melanoma (29%). Most respondents (81%) indicated their disease had responded fully to treatment. The average decile position on our indicator of socio-economic disadvantage did not differ across cancer types, with the average participant in the 6th decile indicating less disadvantage on average. On average, participants had just over one comorbid condition, with higher levels of comorbidity found for those with NHL (mean = 1.42) and prostate cancer (mean = 1.36). Frequency of the specific health conditions are outlined in Supplementary Table 1. While most participants did not report any information needs, 28% reported 1–3 needs and 13% reported 4 or more. Women with breast cancer were most likely to report having 4 or more information needs (18%), compared to only 5% of those with melanoma.

**Table 1:** Demographic characteristics, comorbidity status, disease response and unmet information needs of participants by cancer type

Characteristic	Total		Breast		Colorectal		Melanoma		Non-Hodgkin lymphoma		Prostate		*p* value
	*N*	%	*N*	%	*N*	%	*N*	%	*N*	%	*N*	%	
**Total**	2115		459	21.7	444	21.0	476	22.5	234	11.1	502	23.7	
**Years post diagnosis**													
1 year	759	35.9%	158	34.4	158	35.6	205	43.1	80	34.2	158	31.5	0.004
3 years	637	30.1%	161	35.1	147	33.1	133	27.9	48	20.5	148	29.5	0.001
5 years	719	34.0%	140	30.5	139	31.3	138	29.0	106	45.3	196	39.0	0.000
**Sex**													
Male	1123	53.1	0	0.0	245	55.2	237	49.8	139	59.4	502	100.0	0.000
Female	992	46.9	459	100.0	199	44.8	239	50.2	95	40.6	0	0.0	0.000
**Age**													
< 50 years	297	14.0%	89	19.4	54	12.2	106	22.3	31	13.2	17	3.4	0.000
50–59 years	466	22.0%	133	29.0	81	18.2	109	22.9	50	21.4	93	18.5	0.000
60–69 years	723	34.2%	143	31.2	142	32.0	138	29.0	72	30.8	228	45.4	0.000
70–79 years	452	21.4%	70	15.3	112	25.2	80	16.8	60	25.6	130	25.9	0.000
80 + years	177	8.4%	24	5.2	55	12.4	43	9.0	21	9.0	34	6.8	0.002
**Country of birth**													
Australia	1561	78.8%	336	78.5	298	72.7	401	87.7	164	76.3	362	76.9	0.000
Other	420	21.2%	92	21.5	112	27.3	56	12.3	51	23.7	109	23.1	0.000
**Remoteness index**													
Major city	1429	67.6%	295	64.3	289	65.1	314	66.0	174	74.4	357	71.1	0.018
Inner regional	572	27.0%	138	30.1	127	28.6	139	29.2	49	20.9	119	23.7	0.026
Outer regional/remote	114	5.4%	26	5.7	28	6.3	23	4.8	11	4.7	26	5.2	0.853
**Response to treatment**													
Responded fully to treatment	1627	81.2%	358	82.1	344	81.5	411	89.3	167	77.3	347	74.0	0.000
Treated but still present	161	8.0%	17	3.9	30	7.1	16	3.5	30	13.9	68	14.5	0.000
Not treated	43	2.1%	5	1.1	7	1.7	3	0.7	8	3.7	20	4.3	0.001
Not certain	172	8.6%	56	12.8	41	9.7	30	6.5	11	5.1	34	7.2	0.001
**Information needs**													
No needs	1247	59.0%	240	52.3	248	55.9	324	68.1	126	53.8	309	61.6	0.000
Low needs (1-3)	596	28.2%	135	29.4	129	29.1	127	26.7	76	32.5	129	25.7	0.319
High needs (4 +)	272	12.9%	84	18.3	67	15.1	25	5.3	32	13.7	64	12.7	0.000
**Index of relative socio-economic disadvantage**													
Mean (SD)	2111	6.53 (2.81)	458	6.57 (2.79)	444	6.37 (2.88)	628	6.66 (2.74)	295	6.37 (2.82)	688	6.58 (2.83)	0.519
**Number of longstanding comorbidities**													
Mean (SD)	2115	1.20 (1.29)	459	1.05 (1.17)	444	1.29 (1.40)	476	0.98 (1.19)	234	1.42 (1.37)	502	1.36 (1.29)	0.000

*p* values are from tests of equal proportions for categorical variables and from an analysis of variance for continuous variables

### Health-related quality of life (EQ-5D-5L)

Unstandardised and standardised betas from analyses that regressed total EQ-5D-5L scale on cancer type and demographics, information needs, socio-economic disadvantage and comorbidities variables are shown in Table 2. There were relatively small differences in EQ-5D-5L scores in bivariate analyses across the different demographic and disease specific factors. EQ-5D-5L scores were inversely correlated with number of comorbid conditions (*r* =  − 0.25, *p* < 0.001) but positively correlated with socio-economic position (*r* = 0.10, *p* < 0.01). These relationships were maintained in multivariate analyses, with those 80 years or older (beta =  − 0.07, *p* = 0.013), those with more information needs and those with a greater number of comorbid conditions (beta =  − 0.23, *p* < 0.01) more likely to have lower EQ-5D-5L scores. Residing in areas with less socio-economic disadvantage was associated with higher EQ-5D-5L scores (beta = 0.06, *p* = 0.036).

**Table 2:** Bivariate and multivariate^#^ associations between demographic and disease factors and quality of life (EQ-5D-5L) and Social Difficulties Index *inventory*

Characteristic	EQ-5D-5L index^					Social Difficulties Index^^				
	Mean	Coef	SE	Beta	Multi-variate *p* value	Mean	Coef	SE	Beta	Multi-variate *p* value
**Total**	0.84					3.82				
**Tumour group (*p* value)**										
Breast	0.81	Ref				4.97		Ref		
Colorectal	0.83	0.02	0.02	0.04	0.196	4.54	− 0.44	0.46	− 0.03	0.343
Melanoma	0.89	0.04	0.02	0.09	0.006	2.28	− 1.96	0.46	− 0.14	< 0.001
Non-Hodgkin lymphoma	0.82	0.01	0.02	0.01	0.697	4.67	− 0.74	0.56	− 0.04	0.189
Prostate	0.88	0.06	0.02	0.13	0.002	3.19	− 1.51	0.56	− 0.11	0.007
*Bivariate p value*	< *0.01*					< 0.001				
**Years post diagnosis**										
1 year	0.83	(Ref)				4.24		(Ref)		
3 years	0.85	0.02	0.01	0.04	0.121	3.69	− 0.61	0.35	− 0.05	0.084
5 years	0.86	0.02	0.01	0.04	0.144	3.49	− 0.87	0.35	− 0.07	0.012
*Bivariate p value*	*0.161*					*0.045*				
**Age**										
< 50 years	0.85					4.61				
50–59 years	0.85	− 0.01	0.02	− 0.02	0.628	4.29	− 0.22	0.43	− 0.02	0.612
60–69 years	0.86	0.00	0.02	− 0.01	0.84	3.44	− 0.72	0.45	− 0.06	0.107
70–79 years	0.85	− 0.01	0.02	− 0.02	0.548	3.11	− 1.29	0.52	− 0.08	0.013
80 + years	0.77	− 0.06	0.02	− 0.07	0.013	4.61	− 0.12	0.68	− 0.01	0.858
*Bivariate p value*	*0.005*					< *0.001*				
**Sex**										
Male	0.86	(Ref)				3.23		(Ref)		
Female	0.83	− 0.02	0.01	− 0.04	0.273	4.52	0.38	0.39	0.03	0.325
*Bivariate p value*	< *0.001*					< *0.001*				
**Information needs**										
No needs	0.90	(Ref)				2.36		(Ref)		
Low needs (1–3)	0.81	− 0.07	0.01	− 0.17	< 0.001	4.54	1.74	0.32	0.14	< 0.001
High needs (4 +)	0.72	− 0.17	0.02	− 0.28	< 0.001	8.65	5.23	0.44	0.31	< 0.001
*Bivariate p value*	< *0.001*					< *0.001*				
	Correlation					Correlation				
**Index of relative socio-economic disadvantage (deciles)**	0.10	0.01	0.00	0.06	0.036	− 0.06	− 0.17	0.06	− 0.08	0.002
*Bivariate p value*	< *0.001*					*0.003*				
**Number of longstanding comorbidities**	− 0.25	− 0.05	0.01	− 0.23	< 0.001	0.27	1.18	0.15	0.21	< 0.001
*Bivariate p value*	< *0.001*					< *0.001*				

^Score standardised and range from 0 to 1. Higher scores indicate better quality of life

^^Possible score range: 0–44 higher scores indicate greater social difficulties

^#^Multivariate analyses controlled for country of birth, response to treatment and residential location

### Social difficulties index (SDI)

Overall, average SDI scores were low (Table 2), indicating that on average participants in our study did not regularly experience significant social difficulties. However, there was some variation across different groups. On average, melanoma survivors reported the lowest levels of social difficulties (mean = 2.28) while breast cancer survivors reported the greatest levels (mean = 4.97). The number of comorbid conditions was positively associated with SDI scores (*r* = 0.27, *p* < 0.01); mean SDI scores for those with no comorbid conditions (mean = 2.83) was around half that found for those with three or more comorbid conditions (mean = 6.05). Multivariate patterns of associations were similar to those seen in the bivariate analyses. Having higher information needs increased the likelihood of experiencing higher levels of social difficulties. Comorbidities were positively associated with social difficulties (beta =  0.21, *p* < 0.01) in multivariate analyses, while socio-economic disadvantage was inversely associated (beta =  − 0.08, *p* < 0.01).

### Employment

Not being in paid employment at the time of the survey was assessed in a subset of the sample who were aged under 60 years and in paid employment at diagnosis (*n* = 601). Fifteen percent of this group of participants were not working at the time of the survey. Melanoma survivors were most likely and prostate and colorectal cancer survivors least likely to be working at the time of the survey (Table 3). Not working was more likely for people aged in their 50 s compared to those aged under 50 at diagnosis and for those 5 years post diagnosis compared to those 1 year post diagnosis. In multivariate analyses, respondents who were 5 years post diagnosis (OR = 2.26, 95% CI: 1.09–4.69) and those aged between 50 and 59 years (OR = 3.00, 95% CI: 1.52–5.92) were more likely to not be working at the time of the survey. While decile increase in socio-economic disadvantage reduced the odds of not being in the workforce by 12% (OR = 0.88, 95% CI: 0.79–0.99), every additional comorbid condition increased the odds of not working by 61% (OR = 1.61, 95% CI: 1.16–2.23).

**Table 3:** For people aged under 60 years and in paid employment at diagnosis, bivariate and multivariate associations between demographic and disease factors and not being in paid employment at point of survey

Characteristic	%	Not in paid employment at follow-up			
		Bivariate *p* value	OR	95% CI	
**Total**	14.5				
**Tumour group**					
Breast	16.0		1		
Colorectal	19.3		1.73	0.74	4.04
Melanoma	5.8		0.52	0.20	1.36
Non-Hodgkin lymphoma	17.5		0.88	0.31	2.47
Prostate	20.3	0.013	1.79	0.54	5.91
**Years post diagnosis**					
1 year	9.6		1		
3 years	12.8		1.01	0.45	2.25
5 years	20.1	0.017	2.26	1.09	4.69
**Age at diagnosis**					
< 50 years	7.7		1		
50–59 years	19.4	0.001	3.00	1.52	5.92
**Sex**					
Male	12.6		1		
Female	15.9	0.29	1.94	0.83	4.51
**Information needs**					
No needs	11.1		1		
Low needs (1–3)	13.5		1.64	0.89	3.04
High needs (4 +)	22.5	0.018	2.07	1.00	4.28
**Index of relative socio-economic disadvantage (deciles)**	Mean				
Employed	6.93				
Unemployed	6.28	0.06			
Unit increase (decile)			0.88	0.79	0.98
**Number of longstanding comorbidities**					
Employed	0.70				
Unemployed	1.07	< 0.001			
Increase for every 1 additional comorbid condition			1.61	1.16	2.23

Adjusting for country of birth and response to treatment

### Information needs

Given the associations between information needs and QoL and social difficulties outcomes, we examined multivariate associations between information needs and disease and socio-demographic variables. Survivors of melanoma, older survivors, men and those with fewer comorbid conditions were less likely to report information needs (see Supplementary Table 2).

### Mediation models

Results from mediation analyses are shown in Table 4. Comorbidities partially mediated the effects of socio-economic disadvantage on EQ-5D-5L (IE: Coeff = 0.001; 95% CI: 0.00–0.002) and SDI (IE: Coeff =  − 0.03; 95% CI: − 0.065 to − 0.002) but did not mediate the association between socio-economic disadvantage and work status after cancer for those aged under 60 years. However, while statistically significant, the IE of socio-economic disadvantage through comorbidities for both EQ-5D-5L and SDI was, at 17% and 14% respectively, small. Unmet information needs did not mediate associations between socio-economic disadvantage for EQ-5D-5L, SDI or work status (Table 4).

**Table 4:** Total, direct and indirect effects of socio-economic position on health-related quality of life (EQ-5D-5L), social difficulties index (SDI) and for those working at diagnosis, not returning to work, with number of comorbidities the mediator (unstandardised coefficients (Coeff) or odds ratios (ORs) reported)^^,#,$^

Effect	Variable	EQ-5D-5L index			SDI			Not in work force		
		Coeff	95% CI		Coeff	95% CI		OR	95% CI	
			Lower	Upper		Lower	Upper		Lower	Upper
**Index of relative socio-economic disadvantage (IRSD) mediated through comorbidities**										
**Total**	IRSD-10	0.006	0.001	0.0100	− 0.210	− 0.336	− 0.085	0.89	0.800	0.996
**Direct**	IRSD-10	0.005	0.001	0.009	− 0.180	− 0.302	− 0.068	0.90	0.81	1.003
**Indirect**	Through comorbidities	0.001	0.00	0.002	− 0.030	− 0.065	− 0.002	1.00	0.98	1.015
If significant: % of association mediated		17%			14%			ns		
**Index of relative socio-economic disadvantage (IRSD) mediated through information needs**										
**Total**	IRSD-10	0.006	0.002	0.010	− 0.210	− 0.336	− 0.085	0.893	0.800	0.996
**Direct**	IRSD-10	0.005	0.001	0.009	− 0.190	− 0.309	− 0.071	0.882	0.791	0.984
**Indirect**	Through information needs	0.001	− 0.001	0.002	− 0.021	− 0.061	0.019	1.004	0.989	1.021
If significant: % of association mediated		ns			ns			ns		

^^^*IRSD-10*, index of relative socio-economic disadvantage (deciles)

^#^Analyses adjusted for time since diagnosis, age, gender, cancer type, time since diagnosis, treatment response, country of birth and residential location

^$^Estimates based on 5000 bootstrapped samples

## Discussion

While QoL outcomes were generally very good for all survivors, this study found that older participants, those with multiple comorbid conditions, those from socio-economically disadvantaged areas and those with more information needs were at risk of poorer QoL outcomes. Cancer type also influenced QoL outcomes with higher HRQoL and lower SDI found for people with melanoma and men with prostate cancer compared to women with breast cancer. While comorbidity status partially mediated the associations between socio-economic position and HRQoL and SDI, it did not mediate the association with returning to work. In addition, information needs did not mediate associations between socio-economic position and QoL outcomes. Our results suggest that survivorship care needs to be tailored to individuals given their cancer, age, comorbidity status and socio-economic position.

We used the EQ-5D-5L as our indicator of HRQoL. While this brief non-cancer specific tool allows comparison to population norms, we note it does not provide a detailed assessment of the many dimensions that comprise QoL. Population norms on the EQ-5D-5L for countries similar to Australia in terms of economic development, health system and age of population, range between 0.85 (New Zealand) and 0.90 (Germany) [37]. While average scores on the EQ-5D-5L in our study were slightly lower for those who were 1 year into their survivorship care (0.83), for those three or more years post diagnosis scores were around 0.85, suggesting that for the survivors in our study, HRQoL resembled levels found in the general population. Other studies have found that when non-disease specific indicators of QoL are used, with time, cancer survivors experience a HRQoL similar to age matched controls [21, 38–41]. However, despite the similarity in global HRQoL, detriments in disease specific indicators of QoL including on measures of fatigue, cognitive function, pain and mental health wellbeing tend to persist over time [42]. Similarly mean scores on SDI were generally well below 10, the recommended cut point for indicating that problems with social activities may need further investigation [34]. Despite this, our study found that scores for survivors with three or more comorbid conditions and those with four or more unmet information needs were higher than average. The authors of the SDI suggest that a difference of three or more points on this scale indicates a clinically meaningful difference. Further investigation of survivors with multimorbidity or high information needs is needed with the aim of establishing interventions to reduce social difficulties in these groups.

Our study’s finding that comorbidities reduced HRQoL and increased social difficulties is consistent with a growing body of literature highlighting the role comorbidities have on QoL [21, 22, 43]. Chronic health conditions become more common with age. In Australia, 80% of adults over the age of 65 years have at least one chronic health condition, with arthritis (49%), heart disease (20%) and diabetes (17%) the most common. With 72% of respondents reporting a long-term health condition, comorbidity in our sample is similar to the broader Australian population. Other studies have reported higher levels of comorbidity in cancer survivors; for instance, a recent US study reported that 90% of survivors had a comorbid condition [22], and in a German study of survivors 5 and 10 years post diagnosis, 95% reported comorbidity [20]. Unlike the current study, both included hypertension, with the US study also including high cholesterol. As most chronic health conditions have an independent negative impact on HRQoL [22], findings suggest that quality survivorship care needs to consider the other health conditions that many cancer survivors have.

Our study highlighted the importance of addressing cancer survivors’ unmet needs with lower QoL and higher social difficulties associated with a greater number of unmet information needs. While the cross-sectional nature of our design means we cannot determine whether unmet information needs are a cause or a consequence of lower HRQoL and higher social difficulties, the strength of the associations found suggests the importance of information to improve QoL outcomes. Similar to others [28, 30], we found younger survivors reported more unmet information needs, as did those more recently diagnosed. Socio-economic position was not related to information needs and information needs did not mediate any effect of socio-economic position on our three QoL outcomes. While our results need to be replicated, they suggest that unmet information needs are common across socio-economic groups. We found that a greater number of comorbidities were associated with more unmet information needs. Our findings suggest that quality survivorship programs need to develop strategies to ensure the regular monitoring of information needs of survivors particularly those survivors with comorbidities.

We found small but statistically significant associations between socio-economic disadvantage and our three QoL outcomes in multivariate analyses. This is similar to findings from the USA where survivors from lower socio-economic backgrounds have lower health-related QoL outcomes, even after adjusting for presence of comorbidities [18, 22]. Given known associations between socio-economic position and comorbidity status, our study formally explored the role of comorbidities as a mediator. Our finding that comorbidities mediated some but not all the association between socio-economic position and QoL outcomes suggests that strategies are needed to ensure the health outcomes of people from more socio-economically disadvantaged areas are maximised.

### Strengths and limitations

While the study has several strengths including large sample size, good representation from different cancers and inclusion of survivors at 1, 3 and 5 years post diagnosis, several limitations should be acknowledged. The cross-sectional study design means we cannot determine the direction of associations we identified. While a survivor bias is likely to be evidenced, as the difference in the responses of those 5 years and those 3 years post diagnosis was small we think this bias will be minimal. We did not assess when people were diagnosed with their comorbid condition in relation to their cancer nor did we assess the impact of this condition on respondents. Thus, whether lower QoL scores for people with comorbidities are due to the comorbidity entirely or a combination of cancer and comorbidity is not known. The associations between socio-economic position and QoL outcomes were relatively small. As our use of an area-level indicator of socio-economic position may underestimate this association, more work is needed to understand associations between socio-economic position and QoL outcomes. Our unmet information needs indictor reflected the number of needs rather than the type or degree of need experienced. The lack of nuance in this measure is a limitation. Further work exploring how different types and levels of needs influence QoL is required. Cancer survivors from areas with greater socio-economic disadvantage were underrepresented in our sample, as were survivors from a non-English speaking background. While we controlled for impact of disease response in analyses, we did not control for stage of disease at diagnosis or treatment. Finally, the low response rate must be noted and may give rise to some of the potential biases noted. While a response rate under 50% suggests that caution is needed when extrapolating findings, we note that this response rate is comparable to other patient studies in Australia using cancer registries for recruitment [44, 45].

## Conclusions

Using a population-based cancer registry to identify cancer survivors at 1, 3 and 5 years post diagnosis, we found that survivors from more socio-economically disadvantaged areas, those with more comorbidities and those with more unmet information needs were consistently more likely to report poorer outcomes on three QoL indicators: health-related QoL, social difficulties and employment. Our results show the importance of establishing survivorship care programs that can regularly assess and address the information needs of cancer survivors. Importantly our results show the need to establish survivorship care plans that consider the overall health of the survivor including other health conditions they may have at diagnosis and those that develop subsequently. Being aware of any socio-economic barriers that may reduce access to quality care and implementing strategies to reduce these barriers may assist in achieving optimal outcomes for all cancer survivors.

## Supplementary Information

Below is the link to the electronic supplementary material.

Supplementary file1 (DOCX 18 KB)

## Data Availability

The data that support the findings of this study are available from the corresponding author on reasonable request. The data are not publicly available due to privacy and ethical restrictions.
