# Supplementary material for: Disparities in quality of life, social distress and employment outcomes in Australian cancer survivors
Source: Support Care Cancer. 2022 Mar 12;30(6):5299–309. doi: 10.1007/s00520-022-06914-w (PMC9046289; doi:10.1007/s00520-022-06914-w)
Supplement: Supplementary file 1 — Supplementary file1 (DOCX 18 KB) [file 520_2022_6914_MOESM1_ESM.docx]

**Supplementary Table 1: Prevalence of comorbid conditions (ordered by frequency).**

|  | N | % |
| --- | --- | --- |
| Arthritis | 596 | 28.2 |
| Back problems | 401 | 19.0 |
| Heart disease | 288 | 13.6 |
| Hearing problems | 267 | 12.6 |
| Diabetes | 217 | 10.3 |
| Asthma | 198 | 9.4 |
| Vision problems | 97 | 4.6 |
| Mental health problems | 65 | 3.1 |
| Angina | 54 | 2.6 |
| Kidney problems | 48 | 2.3 |
| Neurological problems | 45 | 2.1 |
| Liver disease | 29 | 1.4 |
| Epilepsy | 20 | 0.9 |
| Alzheimer’s disease | 13 | 0.6 |
| Learning difficulties | 11 | 0.5 |
| Other condition | 187 | 8.8 |
|  |  |  |

**Supplementary Table 2: Association between any unmet needs and disease and sociodemographic characteristics (standard logistic regression)**

|  | Unmet information needs | | |  |
| --- | --- | --- | --- | --- |
|  | OR | 95% Confidence intervals | | p-value |
| **Cancer type** |  |  |  |  |
| Breast | 1 |  |  |  |
| Colorectal | 1.17 | 0.80 | 1.71 | 0.411 |
| Melanoma | 0.58 | 0.40 | 0.84 | 0.004 |
| Non-Hodgkins Lymphoma | 1.54 | 0.97 | 2.44 | 0.066 |
| Prostate | 1.23 | 0.77 | 1.95 | 0.386 |
| **Age at diagnosis** |  |  |  |  |
| < 50 years | 1 |  |  |  |
| 50-59 years | 0.42 | 0.30 | 0.60 | 0.000 |
| 60-69 years | 0.24 | 0.17 | 0.34 | 0.000 |
| 70-79 years | 0.14 | 0.09 | 0.21 | 0.000 |
| 80+ years | 0.09 | 0.05 | 0.17 | 0.000 |
| **Time since diagnosis** |  |  |  |  |
| 1 year |  |  |  |  |
| 3 years | 0.95 | 0.71 | 1.27 | 0.745 |
| 5 years | 0.86 | 0.65 | 1.14 | 0.296 |
| **Index of Relative Socioeconomic Disadvantage (deciles)** |  |  |  |  |
| Increase one decile | 1.01 | 0.75 | 1.35 | 0.966 |
| **Gender** |  |  |  |  |
| male | 1 |  |  |  |
| female | 1.50 | 1.09 | 2.07 | 0.012 |
| **Number of longstanding comorbidities** |  |  |  |  |
| Increase one unit | 1.25 | 1.10 | 1.41 | 0.000 |

Adjusting for country of birth, response to treatment, residential location
